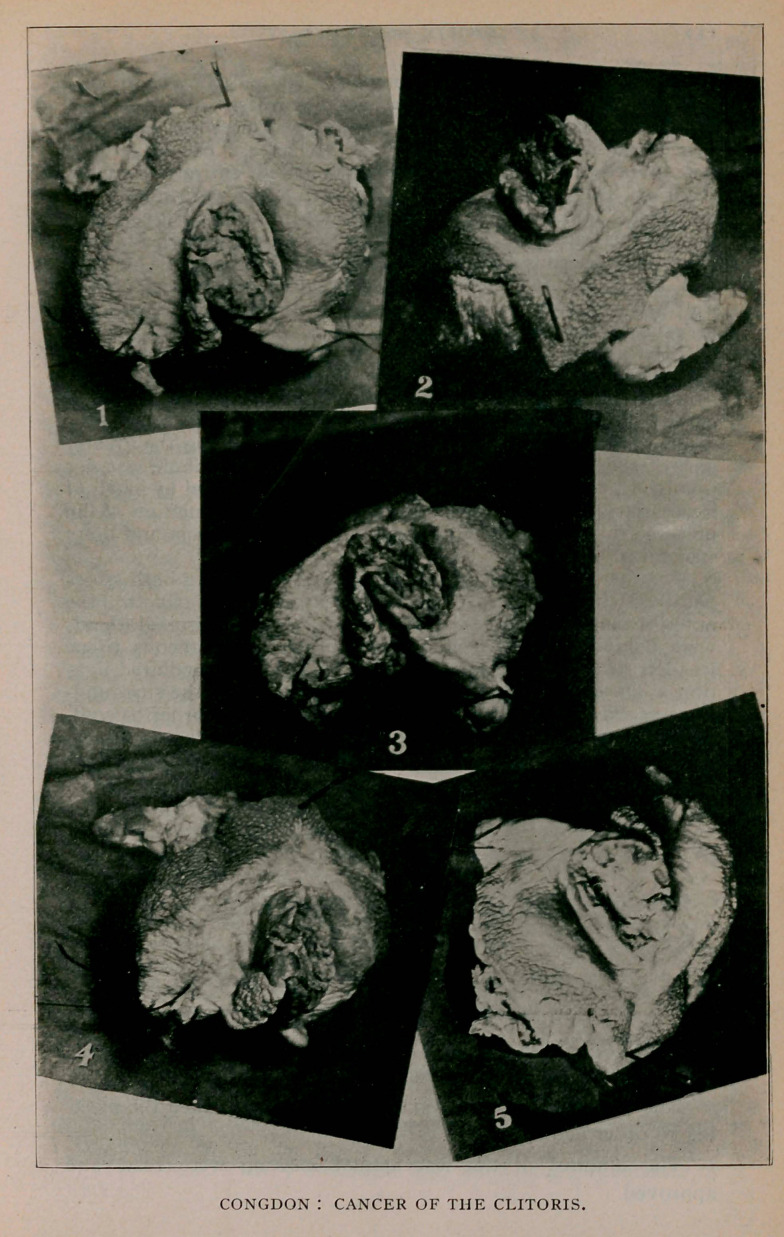# Cancer of the Clitoris1Presented at the Buffalo Academy of Medicine, Section on Obstetrics and Gynecology, April 22, 1902.

**Published:** 1902-09

**Authors:** Charles E. Congdon

**Affiliations:** Buffalo, 1034 Jefferson Street.


### CLINICAL REPORT.

### Cancer of the Clitoris.[1]

BY CHARLES E. CONGDON, M.D., Buffalo.

THE extreme rarity of primary cancer of the clitoris has led me to present this case. I have been able to learn of only two authenticated cases of this kind, and those were reported by Howard Kelly, of Baltimore.

This affection should not be confounded with cancer of the vulva in its latent stages, secondarily involving the clitoris, or with syphilitic papilloma, for these are more common affections.

Mrs. W., aged 69 years; English. Had the various affections of childhood before her fourteenth year, when she began her menstrual function, which continued painlessly and with regularity except when interrupted by pregnancy, to her 45 year, at which time it ceased, she passing through the climacteric period safely. Was married at the age of 18 years, and between her 20th and 39th years bore six living children; the births were normal and easy, instrumentation was not indicated or used. Father died at 68 years, cause unknown; mother at 60 years, from some intestinal trouble, the exact nature of which is unknown. Husband died at 67 years from cancer of the duodenum.

Between her 66th and 67th years, Mrs. W. began to suffer at intervals from itching of the vulva, which she attributed to some kidney or bladder trouble and was treated by various physicians, who failed to examine her and although a very intelligent woman, a warty growth of the clitoris did not, in her opinion, have any significant bearing upon the now incessant itching, burning and ichorous discharge; it was not until sitting upright or locomotion was rendered almost impossible that she submitted to an examination, and was then informed by her physician that an operation was indicated.

She entered my hospital in September, 1901, and upon examination the growth was found to be entirely of the clitoris, which measured 3 by 4 centimeters in diameter, protruding

1. Presented at the Buffalo Academy of Medicine, Section on Obstetrics and Gynecology, April 22, 1902.

CONGDON: CANCER OF THE CLITORIS.

from between the vulva 2 centimeters. The vulva was shriveled and showed evidence of long continued pruritis.

The central portion of the growth was necrotic and gave rise to an offensive sero-purulent discharge. The labiæ were not involved and apparently it originated entirely from the clitoris. The inguinal glands on both sides were noticeably enlarged. Her general health had not suffered until recently, but at this time she complained of sharp pains which radiated from the cancerous clitoris to both inguinal regions. This pain was so severe for several weeks previous to her visit to me, she had required an opiate at night in order to secure the necessary sleep.

Microscopic examination of a fragment of the growth demonstrated it to be carcinoma. The disease was extirpated by an irregular triangular incision beginning at the upper border of the mons veneris and extending in a line downward, somewhat curved away from the growth and including the greater part of the labia on both sides; and while the vestibule was not involved, yet the incision and extirpation included as much of it as was possible without interfering with the function of the urethra; this included all tissues down to the fascia and bony structures.

At the same time an incision was made on both sides parallel with Poupart's ligament, extending nearly to the anterior superior spine of the ilium and the inguinal glands were completely removed; a small bridge of cutaneous tissue was left at the lower angle; a few bleeding points required ligation. The extirpation of the clitoris together with the surrounding structures required a flap-sliding operation in order to completely cover the denuded area. The patient made an uninterrupted recovery and there has been no recurrence of the growth, after eleven months.

1034 Jefferson Street.

## Figures and Tables

**Figure f1:**